# Plasma protein networks linking *APOE ε4* Genotype and Amyloid Status in the Chariot Pro study

**DOI:** 10.1002/alz70861_108757

**Published:** 2025-12-23

**Authors:** Manyue Hu, Oliver J Robinson, Christina M Lill, Anna Matton, Susan Baker, Lefkos T Middleton

**Affiliations:** ^1^ The Ageing Epidemiology (AGE) Research Unit, School of Public Health, Imperial College London, London, England UK

## Abstract

**Background:**

The *APOE ε4* allele is the most validated genetic risk factor for Alzheimer’s disease and elevated amyloid burden is an early and key neuropathological feature of the disease. However, the mechanisms linking *APOE ε4* to amyloid burden remain unclear. We aimed to investigate the organ‐specific protein–protein interaction networks linking *APOE ε4* carriage and amyloid elevation.

**Methods:**

Plasma levels of 7,301 aptamers (6,355 human proteins) were measured in 406 cognitively unimpaired adults (60–86 years, 51% female, 50% with elevated amyloid) from the Chariot PRO study via the SomaScan assay v4.1. Proteins were assigned to nine organ‐system‐based groups using the Human Protein Atlas. For each group, we fitted elastic net models (including age, sex, BMI, and storage duration) to classify *APOE ε4* carriage and amyloid elevation (determined through predefined cut‐offs using PET or CSF measurements) separately, repeating modelling 50 times and retaining proteins selected at least once. STRING‐based protein–protein networks were constructed with the proteins selected for each group on both *APOE ε4* carriage and amyloid elevation classification.

**Results:**

Classification accuracy of both *APOE ε4* carriage status and Amyloid elevation was significantly enhanced by the Inclusion of proteins from the immune or nervous systems in the model: 93.5% vs 93.7% for the former and 62.6% vs 61.0% for the latter. 15 proteins from the immune system (CEACAM3, MYC, CD80, CXCL10, ITGA4, and SPC25) and the nervous system (GAD1, CPLX2, RAB37, ADCYAP1, VAMP2, CCK, CPLX1, MPZ, and COL2A1) emerged as the most powerful discriminators of *APOE ε4* carriage and amyloid elevation with high network centrality. Notably, along with brain enriched proteins included in the nervous system, proteins enriched in immune system emerged as the most powerful discriminators of both *APOE ε4* carriage and amyloid elevation.

**Conclusion:**

By integrating network centrality metrics with machine‐learning classification, we have identified 15 plasma proteins that show strong associations with both *APOE ε4* carriage and amyloid elevation. Our findings may contribute to understanding of biological process linking *APOE ε4* genotype and elevated amyloid burden and highlight the importance of immunological mechanisms in Alzheimer’s pathology.

